# Associations of genetic risk scores based on adult adiposity pathways with childhood growth and adiposity measures

**DOI:** 10.1186/s12863-016-0425-y

**Published:** 2016-08-18

**Authors:** Claire Monnereau, Suzanne Vogelezang, Claudia J. Kruithof, Vincent W. V. Jaddoe, Janine F. Felix

**Affiliations:** 1 The Generation R Study Group, Erasmus MC, University Medical Center Rotterdam, P.O. Box 2040, 3000 CA Rotterdam, The Netherlands; 2 Department of Epidemiology, Erasmus MC, University Medical Center Rotterdam, P.O. Box 2040, 3000 CA Rotterdam, The Netherlands; 3 Department of Pediatrics, Erasmus MC, University Medical Center Rotterdam, P.O. Box 2040, 3000 CA Rotterdam, The Netherlands

**Keywords:** Genome-wide association study, Body mass index, Polymorphism, single nucleotide, Genetics, Pediatrics

## Abstract

**Background:**

Results from genome-wide association studies (GWAS) identified many loci and biological pathways that influence adult body mass index (BMI). We aimed to identify if biological pathways related to adult BMI also affect infant growth and childhood adiposity measures.

**Methods:**

We used data from a population-based prospective cohort study among 3,975 children with a mean age of 6 years. Genetic risk scores were constructed based on the 97 SNPs associated with adult BMI previously identified with GWAS and on 28 BMI related biological pathways based on subsets of these 97 SNPs. Outcomes were infant peak weight velocity, BMI at adiposity peak and age at adiposity peak, and childhood BMI, total fat mass percentage, android/gynoid fat ratio, and preperitoneal fat area. Analyses were performed using linear regression models.

**Results:**

A higher overall adult BMI risk score was associated with infant BMI at adiposity peak and childhood BMI, total fat mass, android/gynoid fat ratio, and preperitoneal fat area (all *p*-values < 0.05). Analyses focused on specific biological pathways showed that the membrane proteins genetic risk score was associated with infant peak weight velocity, and the genetic risk scores related to neuronal developmental processes, hypothalamic processes, cyclicAMP, WNT-signaling, membrane proteins, monogenic obesity and/or energy homeostasis, glucose homeostasis, cell cycle, and muscle biology pathways were associated with childhood adiposity measures (all *p*-values <0.05). None of the pathways were associated with childhood preperitoneal fat area.

**Conclusions:**

A genetic risk score based on 97 SNPs related to adult BMI was associated with peak weight velocity during infancy and general and abdominal fat measurements at the age of 6 years. Risk scores based on genetic variants linked to specific biological pathways, including central nervous system and hypothalamic processes, influence body fat development from early life onwards.

**Electronic supplementary material:**

The online version of this article (doi:10.1186/s12863-016-0425-y) contains supplementary material, which is available to authorized users.

## Background

Childhood overweight and obesity are associated with various adverse short- and long-term consequences, including cardiovascular disease and type 2 diabetes [1–4]. Besides the well-known lifestyle-related risk factors, overweight and obesity have a strong genetic component with heritability estimates from twin studies reported to be up to 80 % [5, 6]. Large genome-wide association studies (GWAS) have identified many single nucleotide polymorphisms (SNPs) associated with body mass index (BMI) in adults [7, 8]. Less is known about the genetic background of BMI in childhood. Three recent studies revealed a total of 15 genetic loci associated with childhood BMI, most of which are also associated with adult BMI [9–11]. We previously reported that a genetic risk score based on 29 SNPs related to adult BMI was associated with infant growth and childhood adiposity measures [12]. A recent GWAS increased the number of adult BMI associated SNPs to 97 [8]. These SNPs are located in or close to genes linked to several biological pathways. In adults especially central nervous system processes seem to play a role [8]. The role of these pathways in body fat development during early life is not known yet. Thus far, GWAS in children did not report any specific biological pathways [11]. Knowledge on the biological pathways influencing BMI from early life onwards may help to better understand the development of overweight and obesity in children.

In this study, we used data from 3,975 children participating in a population-based cohort study to examine the associations of genetic risk scores for adult BMI, both overall and based on specific biological pathways, with infant weight growth patterns and childhood adiposity measures. For comparison, we also examined the associations of genetic risk scores based on the 49 SNPs related with adult waist-hip-ratio (WHR) and on the 15 SNPs associated with childhood BMI with the same infant and childhood outcomes [11, 13].

## Methods

### Study design and population

This study was embedded in the Generation R Study, a population-based, prospective cohort study from fetal life onwards in Rotterdam, the Netherlands [14]. All pregnant women with an expected delivery date between April 2002 and January 2006 and living in Rotterdam were asked to participate. The study was approved by the local Medical Ethical Committee and written consent was obtained for each participating child. GWA scans were available for 59 % of all children (*N* = 5,732) [15]. The Generation R Study is a multi-ethnic cohort. Participants of European origin constitute the largest ethnic group (56 %), and the largest other groups are Surinamese (9 %), Turkish (7 %) and Moroccan (6 %) [14]. Our present study included all singleton live births with GWA data and information on at least one of the outcomes of interest (*N* = 4,151). A participant flowchart is shown in Additional file 1: Figure S1.

### Genetic variants and risk scores

DNA was isolated from cord blood or, in a small minority of children with missing cord blood samples, at 6 years of age. For genome-wide association analysis the Illumina 610 and 660 W Quad platforms were used [16]. Stringent quality checks were performed in which individuals with low sample call rates (<97.5 %) or sex mismatches were excluded. Imputation of genotypes to the cosmopolitan panel of HapMap ii (release 22) was done using MACH software [17, 18]. Prior to imputation, we excluded SNPs with a high level of missing data (SNP call rate <98 %), significant deviations from Hardy-Weinberg equilibrium (*P* < 1*10^−6^), or low minor allele frequencies (<0.1 %). Information about the SNPs of interest for the current study was extracted from the GWAS dataset. The average imputation quality for all SNPs included in this study was 0.96, ranging from 0.55 to 1.00, demonstrating overall good imputation. For 93 out of the 97 known BMI SNPs information was available in our GWA dataset. We used proxies (*R*^2^ > 0.96, D’ = 1) for the remaining four BMI SNPs: rs13012571 was used as a proxy for rs13021737, rs1978487 for rs9925964, rs6445197 for rs2365389, and rs9636202 for rs17724992. Thus, the total number of SNPs used in the analysis was 97 (Additional file 2: Table S1). These SNPs were combined into weighted BMI genetic risk scores (see below). The same procedure was used for the 49 WHR and 15 child BMI SNPs [11, 13]. For 46 of the 49 WHR SNPs information was available in the GWA dataset. Rs4607103 was used as a proxy for rs2371767 (*R*^2^ = 0.90, D’ = 1). For the WHR SNPs rs8042543 and rs6556301 no perfect proxy was available leading to a total number of SNPs of 47 for WHR. For all but one SNPs identified for childhood BMI, information was available in our dataset. We used rs3751812 as a proxy for rs1421085 (*R*^2^ = 0.93, D’ = 0.97) (Additional file 2: Table S1).

In the paper on adult BMI, the 97 adult BMI SNPs were categorized into pathway categories. The authors performed a literature search, which brought about 405 genes within 500 kb on either side and with *r*^2^ > 0.2 of the 97 SNPs [8]. Based on their biological function, these genes were then catagorized into 28 pathways. We used this same categorization, but we excluded categories consisting of one SNP only. For each pathway category, we combined SNPs into a weighted genetic risk score. Some SNPs were included in more than one category based on their biological function (Additional file 3: Table S2). The number of overlapping SNPs between the biological categories is shown in Additional file 4: Table S3. As a comparison we ran a pathway analysis using QIAGEN’s Ingenuity® Pathway Analysis software (IPA) (IPA®, QIAGEN Redwood City,www.qiagen.com/ingenuity).

### Infant weight growth and childhood general and abdominal adiposity

We used repeated growth measurements to derive infant peak weight velocity (PWV), BMI at adiposity peak (BMIAP) and age at adiposity peak (AGEAP), as described previously [19–23]. Briefly, the Reed1 model was used for boys and girls separately, to obtain PWV during infancy. BMIAP and AGEAP were obtained by fitting a cubic mixed effects model on log (BMI) from 2 weeks to 1.5 years of age while adjusting for sex.

At the median age of 6.0 years (95 % range, 5.7, 7.4) we measured general and abdominal adiposity measures as described in detail previously [24]. Briefly, BMI (kg/m^2^) was calculated from height and weight measured without shoes and heavy clothing. Total, android, and gynoid fat mass were measured by Dual-energy X-ray absorptiometry (DXA) (iDXA, GE-Lunar, 2008, Madison, WI, USA) [24]. Total fat mass (kg) was calculated as a percentage of total body weight (kg). Android/gynoid fat ratio provides the ratio of central body fat distribution in the abdomen (android fat) and hip (gynoid fat) regions [25]. Preperitoneal fat area, which is a measure of visceral abdominal fat, was measured by abdominal ultrasound [24, 26, 27].

### Statistical analysis

We constructed a weighted genetic risk score combining the 97 adult BMI SNPs summing the number of outcome increasing risk alleles from the GWA dosage data, weighted using effect estimates of risk increasing alleles in adults. The risk score was rescaled to standard deviation scores (SDS, (observed value-mean)/standard deviation (SD)). Similarly, we constructed genetic risk scores based on SNPs involved in 28 different biological categories, and based on 47 adult WHR SNPs and 15 childhood BMI SNPs. For the biological categories and the WHR SNPs, we used the effect estimates from the original papers as weights [8, 11, 13]. For the 15 childhood SNPs, weights were obtained from the GWAS meta-analysis without the Generation R data [11]. We used linear regression analyses to examine the associations of the risk scores with PWV, BMIAP, and AGEAP in infancy, and BMI, total fat mass percentage, android/gynoid fat ratio, and preperitoneal fat area in childhood. The variance explained by the risk scores was considered to be the increase in the unadjusted R^2^ between the model containing all covariates and the risk score or separate SNPs, and the same model without the risk score. For all analyses, we natural logarithm transformed total fat mass, android/gynoid fat ratio, and preperitoneal fat area to obtain a normal distribution. Standard deviation scores were created for all outcome measures to allow comparison of effect estimates. For BMI, age-adjusted SD scores were created using the Dutch reference growth curves (Growth Analyzer 3.0, Dutch Growth Research Foundation, Rotterdam, the Netherlands) [12]. To enable comparison with our current risk scores, we rescaled the previously published 29 adult BMI SNPs risk score to SD scores. All models were adjusted for sex plus the first four principal components from the genetic data to adjust for ethnic background [28]. Models for general and abdominal adiposity measures were additionally adjusted for age except for BMI which was already age adjusted. Models for total fat mass, android/gynoid fat ratio, and preperitoneal fat area were additionally adjusted for height. [24] We also tested whether the associations of the child and adult BMI risk scores with the childhood adiposity outcomes were explained by infant growth by adding PWV and BMIAP separately to the regression models. For the analyses of the 28 biological pathways, we applied Bonferroni correction and considered a *p*-value of <0.0018 (0.05/28) as significant. All analyses were performed using the Statistical Package for the Social Sciences version 21.0 for Windows (SPSS; IBM, Chicago, IL, USA).

## Results

### Characteristics of the study population

Characteristics of all children are listed in Table 1. The children had a median age of 6.0 years (95 % range 5.7, 7.4). The median BMI at that age was 15.8 (95 % range 13.7, 21.2).

**Table 1:** Characteristics of the study population

Characteristics	Full group (*N* = 3,975)	European (*N* = 2,566)	Turkish (*N* = 300)	Surinamese (*N* = 287)	Moroccan (*N* = 234)	Other (*N* = 588)
Birth						
Boys	50.2 %	49.6 %	53.7 %	53.3 %	50.4 %	49.2 %
Gestational age at birth (weeks)^a^	40.1 (36.4; 42.3)	40.3 (33.3; 42.0)	40.0 (36.2; 42.3)	39.7 (35.7; 42.0)	40.6 (36.4; 42.2)	40.0 (36.4; 42.1)
Weight at birth (grams)	3458 (514)	3506 (514)	3402 (480)	3238 (536)	3496 (426)	3379 (506)
Infant						
Peak weight velocity (kg/year)	12.2 (2.1)	12.0 (2.0)	13.1 (2.4)	12.5 (2.2)	12.6 (2.1)	12.4 (2.2)
Body mass index at adiposity peak (kg/m^2^)	17.6 (0.8)	17.5 (0.8)	17.9 (0.9)	17.5 (0.9)	17.8 (0.8)	17.7 (0.8)
Age at adiposity peak (years)	0.7 (0.04)	0.7 (0.04)	0.7 (0.04)	0.7 (0.04)	0.7 (0.04)	0.7 (0.04)
Childhood						
Age at visit (years)^a^	6.0 (5.7; 7.8)	6.0 (5.7; 7.5)	6.1 (5.7; 7.7)	6.1 (5.5; 8.2)	6.1 (5.7; 8.3)	6.1 (5.7; 8.2)
Height (cm)	119.6 (6.0)	119.5 (5.6)	119.0 (5.7)	119.9 (7.0)	119.1 (5.9)	120.1 (4.9)
Weight (kg)	23.3 (4.2)	22.9 (3.6)	24.5 (5.3)	23.5 (5.3)	23.9 (4.1)	24.0 (4.9)
Body mass index (kg/m^2^)^a^	15.8 (13.7; 21.3)	15.7 (13.7; 19.8)	16.6 (13.6; 24.2)	15.7 (13.2; 23.3)	16.4 (14.0; 22.0)	16.2 (13.6; 22.0)
Total fat mass percentage^a^	24.0 (16.3; 38.6)	23.5 (16.4; 36.4)	26.6 (18.3; 43.5)	24.1 (14.8; 41.4)	25.9 (17.8; 39.9)	24.3 (15.9; 39.4)
Android-gynoid fat ratio^a^	0.2 (0.2; 0.4)	0.2 (0.2; 0.4)	0.3 (0.2; 0.5)	0.2 (0.2; 0.5)	0.2 (0.2; 0.4)	0.2 (0.1; 0.4)
Preperitoneal fat area (cm^2^)^a^	0.4 (0.2; 1.2)	0.4 (0.2; 1.0)	0.5 (0.2; 1.9)	0.4 (0.2; 1.7)	0.4 (0.2; 1.6)	0.4 (0.2; 1.3)
Overweight (%)^b^	12.9	10.5	23.7	11.8	19.7	17.8
Obese (%)^b^	4.1	2.1	11.0	8.0	7.7	6.1

*N* = 3,975

Values are means (standard deviations) unless otherwise specified

^a^Median (95 % range)

^b^The IOTF-classification was used to define overweight and obesity [41]

### Infant weight growth patterns

**Fig. 1:**
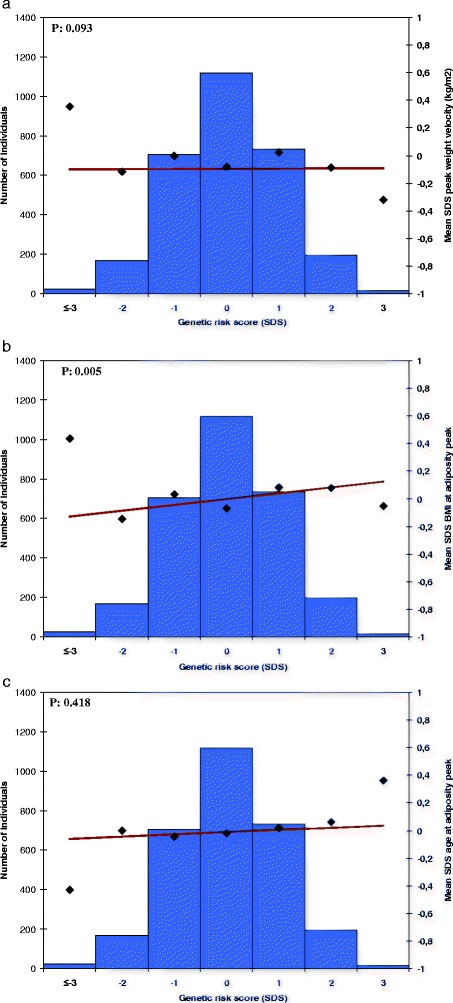
Association of adult body mass index genetic risk score with infant growth measures (*N* = 2,955). The *x* axis represents the categories of the risk score (overall sum of risk alleles, weighted by previously reported effect estimates, rescaled to SDS. The risk score ranged from −4 to 3 SDS and was rounded to the nearest integer for clarity of presentation. The right *y* axis shows mean SDS and corresponds to the dots and the line representing the regression of the mean SDS values for each category of the risk score. The *y* axis on the left corresponds to the histogram representing the number of individuals in each risk-score category. *P*-value is based on the continuous risk score, as presented in Table 2. Graphs represent; **a** peak weight velocity, **b** BMI at adiposity peak, and **c** age at adiposity peak

The overall adult BMI genetic risk score was associated with BMIAP (Table 2; Fig. 1a-c), but not with other infant weight growth measures. BMIAP increased by 0.048 SDS (95 % confidence interval (CI) 0.015, 0.081) per SD increase in the genetic risk score. Of the 28 adult BMI genetic risk scores based on biological pathways, only the membrane proteins pathway genetic risk score was associated with PWV (*p*-value <0.002). Effect estimates for the unweighted and weighted 97 adult BMI SNPs risk scores were similar (Additional file 5: Table S4). As a comparison, the overall adult WHR genetic risk score was not associated with any infant growth measure (Table 2; Additional file 6: Figure S2a-c), whereas the childhood BMI genetic risk score was associated with PWV and BMIAP (0.048 SDS (95 % CI 0.016, 0.079) and 0.051 SDS (0.017, 0.084), respectively, per SD increase in the genetic risk score) (Table 2; Additional file 7: Figure S3a-c). The genetic risk score based on 29 adult BMI SNPs showed lower effect estimates per SD increase than our 97 SNPs adult BMI risk score for PWV and BMIAP, and a higher effect estimate for AGEAP, although none of the associations were significant for the 29 SNP genetic risk score (Additional file 8: Table S5). The largest variance explained by the adult BMI and pathway risk scores was obtained for the membrane proteins pathway with PWV (0.33 %) (Additional file 9: Table S6).

**Table 2:** Associations of BMI, WHR, and childhood BMI genetic risk scores with infant growth (*N* = 2,955)^a^

Risk score (number of SNPs in risk score)	Peak weight velocity^b^		BMI at adiposity peak^b^		Age at adiposity peak^b^	
	Beta (CI 95 %)	*P*-value	Beta (CI 95 %)	*P*-value	Beta (CI 95 %)	*P*-value
Main risk scores*						
Adult BMI (*N* = 97)	0.027 (−0.004; 0.058)	0.093	0.048 (0.015; 0.081)	**0.005**	0.015 (−0.021; 0.051)	0.418
Secondary risk scores						
Adult WHR (*N* = 47)	−0.022 (−0.054; 0.010)	0.180	−0.010 (−0.044; 0.025)	0.587	−0.016 (−0.053; 0.022)	0.411
Child BMI (*N* = 15)	0.038 (0.007; 0.070)	**0.018**	0.039 (0.006; 0.073)	**0.023**	0.027 (−0.010; 0.063)	0.153
Adult BMI pathway genetic risk scores**						
Neuronal						
Neuronal developmental processes (*N* = 29)	0.036 (0.003; 0.070)	0.031	0.049 (0.013; 0.084)	0.007	−0.020 (−0.058; 0.019)	0.311
Neurotransmission (*N* = 10)	−0.009 (−0.040; 0.022)	0.558	−0.001 (−0.034; 0.032)	0.948	0.002 (−0.034; 0.038)	0.901
Hypothalamic expression and regulation (*N* = 13)	0.001 (−0.030; 0.033)	0.932	0.008 (−0.025; 0.042)	0.637	0.023 (−0.013; 0.059)	0.203
Neuronal expression (*N* = 12)	−0.034 (−0.065; −0.003)	0.034	−0.010 (−0.044; 0.024)	0.559	0.026 (−0.010; 0.062)	0.159
Lipid biosynthesis and metabolism (*N* = 10)	0.002 (−0.030; 0.033)	0.918	0.006 (−0.028; 0.040)	0.358	0.020 (−0.017; 0.056)	0.291
Bone development (*N* = 9)	0.017 (−0.014; 0.048)	0.290	0.017 (−0.017; 0.050)	0.336	0.001 (−0.035; 0.037)	0.957
Signaling						
MAPK1/extracellular signal-regulated kinases (*N* = 9)	0.009 (−0.022; 0.040)	0.579	0.008 (−0.025; 0.042)	0.625	0.011 (−0.025; 0.047)	0.534
JAK (*N* = 2)	−0.007 (−0.038; 0.025)	0.679	−0.005 (−0.038; 0.029)	0.779	−0.006 (−0.042; 0.030)	0.750
CyclicAMP (*N* = 5)	−0.020 (−0.052; 0.013)	0.233	0.019 (−0.015; 0.054)	0.368	−0.016 (−0.053; 0.021)	0.391
WNTSignaling (*N* = 6)	0.033 (0.001; 0.064)	0.041	0.017 (−0.016; 0.051)	0.311	0.019 (−0.017; 0.055)	0.293
G-protein coupled receptor						
Notch signaling (*N* = 2)	0.010 (−0.021; 0.041)	0.531	0.009 (−0.024; 0.043)	0.581	0.012 (−0.024; 0.048)	0.508
Mitochondrial (*N* = 8)	0.010 (−0.023; 0.043)	0.559	0.004 (−0.032; 0.039)	0.840	0.039 (0.001; 0.077)	0.046
Retinoic acid receptors (*N* = 6)	0.019 (−0.013; 0.050)	0.245	0.025 (−0.009; 0.058)	0.144	0.019 (−0.017; 0.055)	0.308
Endocytosis/exocytosis (*N* = 14)	0.004 (−0.027; 0.036)	0.778	0.005 (−0.028; 0.038)	0.776	0.007 (−0.029; 0.043)	0.699
Eye-related (*N* = 5)	0.010 (−0.022; 0.042)	0.548	0.010 (−0.025; 0.045)	0.567	−0.030 (−0.067; 0.007)	0.116
Tumorigenesis (*N* = 11)	0.018 (−0.015; 0.050)	0.285	0.018 (−0.017; 0.052)	0.320	−0.001 (−0.038; 0.036)	0.954
Apoptosis (*N* = 13)	0.027 (−0.004; 0.059)	0.087	0.018 (−0.016; 0.052)	0.294	0.033 (−0.004; 0.069)	0.077
Membrane proteins (*N* = 12)	0.057 (0.025; 0.088)	3.88*10 ^**−4**^	0.048 (0.015; 0.082)	0.005	0.028 (−0.008; 0.065)	0.124
Hormone metabolism/regulation (*N* = 4)	−0.009 (−0.041; 0.022)	0.564	−0.009 (−0.042; 0.025)	0.610	0.010 (−0.027; 0.046)	0.604
Purine/pyrimidine cycle (*N* = 4)	0.009 (−0.022; 0.041)	0.557	0.039 (0.006; 0.073)	0.023	−0.025 (−0.061; 0.011)	0.178
Monogenic obesity/energy homeostasis (*N* = 9)	−0.013 (−0.045; 0.018)	0.406	−0.014 (−0.048; 0.020)	0.413	0.026 (−0.011; 0.062)	0.168
Immune system (*N* = 15)	0.045 (0.014; 0.076)	0.005	0.049 (0.015; 0.082)	0.004	−0.003 (−0.039; 0.033)	0.868
Limb development (*N* = 3)	0.018 (−0.014; 0.049)	0.267	0.022 (−0.011; 0.056)	0.195	0.001 (−0.035; 0.037)	0.945
Ubiquitin pathways (*N* = 6)	−0.006 (−0.038; 0.025)	0.684	0.007 (−0.027; 0.040)	0.693	−0.025 (−0.061; 0.011)	0.168
Glucose homeostasis/diabetes (*N* = 11)	0.023 (−0.009; 0.054)	0.160	0.021 (−0.013; 0.055)	0.219	0.026 (−0.010; 0.063)	0.156
Cell cycle (*N* = 23)	0.008 (−0.023; 0.039)	0.611	0.011 (−0.023; 0.044)	0.538	−0.001 (−0.037; 0.035)	0.959
DNARepair						
Nuclear trafficking (*N* = 4)	−0.015 (−0.047; 0.017)	0.362	−0.023 (−0.057; 0.011)	0.187	−0.032 (−0.068; 0.005)	0.092
Muscle biology (*N* = 6)	−0.011 (−0.043; 0.020)	0.479	−0.0003 (−0.034; 0.033)	0.985	0.014 (−0.022; 0.050)	0.446

* Bold font indicates *P*-value < 0.05. ** Bold font indicates significant after Bonferroni correction for the 28 pathways (*p*-value < 0.0018)

^a^Analyses were performed in children with complete data on genetic variants, at least one outcome under study, and covariates

^b^Values are linear regression coefficients for models adjusted for sex and the first four genetic principal components and represent the difference in standard deviation scores of the outcome measures for each additional average risk allele in the risk scores

### General and abdominal adiposity at school-age

**Fig. 2:**
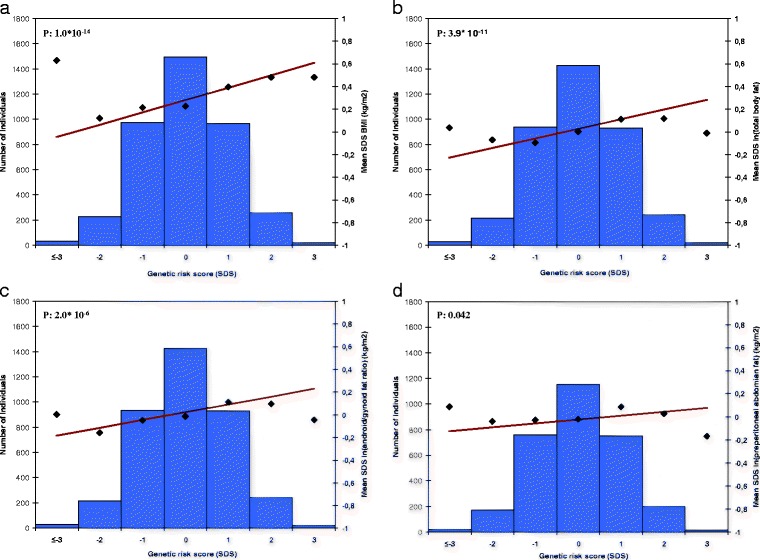
Association of adult body mass index genetic risk score with childhood adiposity measures (*N* = 3975). The *x* axis represents the categories of the risk score (overall sum of risk alleles, weighted by previous reported effect estimates, rescaled to SDS. The risk score ranged from −4 to 3 SDS and was rounded to the nearest integer for clarity of presentation). The right *y* axis shows the mean SDS and corresponds to the dots and a line representing the regression line of the mean SDS values for each category of the risk score. The *y* axis on the left corresponds to the histogram representing the number of individuals in each risk-score category. *P*-value is based on the continuous risk score, as presented in Table 3. Graph **a-d** represent; **a** BMI in kg/m^2^, **b** ln (fat mass percentage), **c** ln (android/gynoid fat ratio), and **d** ln (preperitoneal fat area)

The overall adult BMI genetic risk score was associated with all childhood general and abdominal adiposity measures. For each SD increase in the genetic risk score, childhood BMI increased by 0.112 SDS (95 % CI 0.084, 0.141), total fat mass increased by 0.092 SDS (95 % CI 0.065, 0.119), android/gynoid fat ratio increased by 0.077 SDS (95 % CI 0.045, 0.108), and increased preperitoneal fat area by 0.034 SDS (95 % CI 0.001, 0.066) (Table 3; Fig. 2a-d). Effect estimates for the unweighted and weighted 97 adult BMI SNPs risk scores were similar (Additional file 5: Table S4). Addition of PWV to the regression models did not materially change the effect estimates for the association of the BMI risk scores with BMI, total fat mass percentage, and android/gynoid fat ratio. However, the effect estimate for the association of the adult BMI risk score with childhood preperitoneal fat area was no longer significant. We observed similar findings when we added BMIAP instead of PWV to these regression models. However, the effects on the associations of the BMI risk scores with BMI and total fat mass were somewhat larger. Effect estimates for the associations of the child BMI risk score with BMI and total fat mass were 10–15 % lower after additional adjustment for PWV. Effect estimates for android/gynoid fat ratio and preperitoneal fat area did not materially change. We observed similar findings after additional adjustment for BMIAP (Additional file 10: Table S7 and Additional file 11: Table S8).

**Table 3:** Associations of BMI, WHR, and childhood BMI genetic risk scores with childhood adiposity (*N* = 3,975)^a, b^

Risk score (number of SNPs in risk score)	Body mass index^c^		Total fat mass^c,d,e^		Android/gynoid ratio^c,d,e^		Preperitoneal fat area^c,d,e^	
	Beta (CI 95 %)	*P*-value	Beta (CI 95 %)	*P*-value	Beta (CI 95 %)	*P*-value	Beta (CI 95 %)	*P*-value
Main risk scores*								
Adult BMI (*N* = 97)	0.112 (0.084; 0.141)	1.01*10 ^**−14**^	0.092 (0.065; 0.119)	3.89*10 ^**−11**^	0.077 (0.045; 0.108)	2.00*10 ^**−6**^	0.034 (0.001; 0.066)	**0.042**
Secondary risk scores								
Adult WHR (*N* = 47)	−0.012 (−0.042; 0.017)	0.405	−0.012 (−0.040 0.016)	0.402	0.073 (0.041; 0.105)	8.00*10 ^**−6**^	0.029 (−0.004; 0.061)	0.088
Child BMI (*N* = 15)	0.091 (0.063; 0.119)	3.43*10 ^**−10**^	0.073 (0.046; 0.100)	1.40*10 ^**−7**^	0.081 (0.050; 0.112)	3.75*10 ^**−7**^	0.038 (0.006; 0.070)	**0.020**
Adult BMI pathway genetic risk scores**								
Neuronal								
Neuronal developmental processes (*N* = 29)	0.018 (0.014; 0.023)	2.25*10-5	0.032 (0.003; 0.061)	0.031	0.038 (0.004; 0.071)	0.029	0.008 (−0.026; 0.042)	0.654
Neurotransmission (*N* = 10)	0.013 (−0.015; 0.042)	0.370	−0.003 (−0.030; 0.024)	0.827	0.002 (−0.029; 0.034)	0.876	−0.009 (−0.040; 0.023)	0.595
Hypothalamic expression and regulation (*N* = 13)	0.099 (0.071; 0.128)	5.81*10 ^**−12**^	0.089 (0.062; 0.115)	1.29*10 ^**−10**^	0.080 (0.049; 0.111)	5.30*10 ^**−7**^	0.041 (0.009; 0.073)	0.013
Neuronal expression (*N* = 12)	0.017 (−0.012; 0.046)	0.240	0.020 (−0.008; 0.047)	0.165	0.036 (0.004; 0.068)	0.027	0.009 (−0.023; 0.041)	0.583
	0.023 (−0.005; 0.052)	0.112	0.013 (−0.014; 0.041)	0.341	0.016 (−0.016; 0.048)	0.320	−0.001 (−0.033; 0.032)	0.972
Bone development (*N* = 9)	0.018 (−0.011; 0.064)	0.226	0.006 (−0.021; 0.033)	0.656	0.015 (−0.016; 0.047)	0.340	0.004 (−0.028; 0.036)	0.811
Signaling								
MAPK1/extracellular signal-regulated kinases (*N* = 9)	0.034 (0.006; 0.062)	0.018	0.037 (0.010; 0.064)	0.008	0.023 (−0.008; 0.054)	0.149	0.014 (−0.017; 0.046)	0.378
JAK (*N* = 2)	0.033 (0.005; 0.062)	0.023	0.020 (−0.007; 0.047)	0.150	0.012 (−0.020; 0.043)	0.457	0.007 (−0.025; 0.039)	0.676
CyclicAMP (*N* = 5)	0.046 (0.017; 0.075)	0.002	0.052 (0.024; 0.079)	2.75*10 ^**−4**^	0.039 (0.006; 0.071)	0.019	0.026 (−0.007; 0.058)	0.123
WNTSignaling (*N* = 6)	0.058 (0.030; 0.087)	6.10*10 ^**−5**^	0.029 (0.002; 0.057)	0.034	0.039 (0.007; 0.070)	0.016	0.032 (0.000; 0.064)	0.047
G-protein coupled receptor								
Notch signaling (*N* = 2)	−0.027 (−0.056; 0.001)	0.059	−0.028 (−0.055; 0.000)	0.046	−0.028 (−0.059; 0.003)	0.075	−0.028 (−0.060; 0.003)	0.080
Mitochondrial (*N* = 8)	0.040 (0.010; 0.070)	**0.009**	0.041 (0.012; 0.069)	0.005	−0.002 (−0.035; 0.031)	0.905	−0.003 (−0.037; 0.031)	0.877
Retinoic acid receptors (*N* = 6)	0.045 (0.017; 0.074)	0.002	0.037 (0.010; 0.065)	0.007	0.016 (−0.015; 0.047)	0.313	0.017 (−0.015; 0.049)	0.293
Endocytosis/exocytosis (*N* = 14)	−0.012 (−0.041; 0.016)	0.400	−0.003 (−0.030; 0.024)	0.840	−0.021 (−0.053; 0.010)	0.178	−0.020 (−0.051; 0.012)	0.218
Eye-related (*N* = 5)	0.012 (−0.016; 0.041)	0.398	0.015 (−0.012; 0.043)	0.276	−0.012 (−0.044; 0.020)	0.456	0.003 (−0.029; 0.035)	0.845
Tumorigenesis (*N* = 11)	0.041 (0.012; 0.070)	0.006	0.020 (−0.008; 0.048)	0.161	0.017 (−0.016; 0.049)	0.312	0.013 (−0.020; 0.046)	0.431
Apoptosis (*N* = 13)	0.025 (−0.003; 0.054)	0.084	0.020 (−0.007; 0.047)	0.151	−0.008 (−0.039; 0.024)	0.621	−0.036 (−0.068; −0.004)	0.028
Membrane proteins (*N* = 12)	0.075 (0.046; 0.103)	2.44*10 ^**−7**^	0.044 (0.017; 0.071)	**0.002**	0.059 (0.028; 0.090)	1.93*10 ^**−4**^	0.011 (−0.021; 0.044)	0.495
Hormone metabolism/regulation (*N* = 4)	0.021 (−0.008; 0.049)	0.161	0.043 (0.015; 0.070)	0.002	0.026 (−0.005; 0.057)	0.103	0.004 (−0.028; 0.036)	0.812
Purine/pyrimidine cycle (*N* = 4)	0.013 (−0.016; 0.041)	0.379	0.004 (−0.023; 0.031)	0.762	−0.017 (−0.048; 0.014)	0.285	−0.007 (−0.038; 0.024)	0.661
Monogenic obesity/energy homeostasis (*N* = 9)	0.074 (0.045; 0.102)	4.74*10 ^**−7**^	0.068 (0.041; 0.095)	1.00*10 ^**−6**^	0.065 (0.034; 0.096)	5.00*10 ^**−5**^	0.030 (−0.003; 0.062)	0.072
Immune system (*N* = 15)	0.045 (0.017; 0.074)	0.002	0.037 (0.010; 0.065)	0.008	0.021 (−0.011; 0.052)	0.193	−0.008 (−0.040; 0.024)	0.620
Limb development (*N* = 3)	0.035 (0.007; 0.064)	0.015	0.024 (−0.006; 0.049)	0.125	0.028 (−0.003; 0.060)	0.076	0.004 (−0.028; 0.036)	0.794
Ubiquitin pathways (*N* = 6)	−0.007 (−0.036; 0.021)	0.617	0.006 (−0.021; 0.034)	0.656	−0.011 (−0.043; 0.020)	0.483	−0.015 (−0.047; 0.017)	0.359
Glucose homeostasis/diabetes (*N* = 11)	0.050 (0.021; 0.079)	**0.001**	0.023 (−0.004; 0.051)	0.096	0.042 (0.011; 0.074)	0.008	0.009 (−0.023; 0.042)	0.575
Cell cycle (*N* = 23)	0.044 (0.016; 0.073)	0.002	0.047 (0.019; 0.074)	**0.001**	0.024 (−0.007; 0.055)	0.135	0.007 (−0.025; 0.039)	0.684
DNARepair								
Nuclear trafficking (*N* = 4)	−0.005 (−0.034; 0.023)	0.716	−0.009 (−0.036; 0.018)	0.518	−0.004 (−0.036; 0.028)	0.804	−0.008 (−0.041; 0.024)	0.608
Muscle biology (*N* = 6)	0.048 (0.020; 0.077)	**0.001**	0.029 (0.002; 0.057)	0.038	0.025 (−0.007; 0.056)	0.127	0.022 (−0.010; 0.054)	0.181

* Bold font indicates *P*-value < 0.05. ** Bold font indicates significant after Bonferroni correction for the 28 pathways (*p*-value < 0.0018)

^a^Analyses were performed in children with complete data on genetic variants, at least one outcome under study, and covariates

^b^Values are linear regression coefficients for models adjusted for sex and the first four genetic principal components and represent the difference in standard deviation scores of the outcome measures for each additional average risk allele in the risk scores

^c^Values are additionally adjusted for age

^d^Values are additionally adjusted for height

^e^Regression coefficients are based on standard deviation scores of ln-transformed outcome measures

Of the 28 adult BMI genetic risk scores based on the biological pathways, those based on neuronal developmental processes, hypothalamic expression and regulation, WNT-signaling, membrane proteins, monogenic obesity/energy homeostasis, glucose homeostasis/diabetes, and muscle biology were associated with childhood BMI (all *p*-values <0.0018). Genetic risk scores based on hypothalamic expression and regulation, cyclicAMP, monogenic obesity/energy homeostasis, and cell cycle were associated with total fat mass, whereas for android/gynoid fat ratio only the genetic risk scores based on hypothalamic expression and regulation, membrane proteins, and monogenic obesity/energy homeostasis show significant associations (all *p*-values <0.0018). None of the pathways were associated with preperitoneal fat area (Table 3). We based our pathway risk scores on these biological categories to keep our analysis as close as possible to the analysis of the original paper as possible [8]. As a comparison, we also ran a pathway analysis using IPA. Results were comparable regarding the major categories (eg. neurological development and function, cell cycle, lipid metabolism, apoptosis). However, the IPA software showed a larger subdivision with 74 different pathways instead of 28 as suggested by the GIANT consortium (Additional file 12, Table S9). The overall adult WHR genetic risk score was only associated with android/gynoid fat ratio (Table 3; Additional file 13: Figure S4a-d). The childhood BMI genetic risk score was associated with all childhood adiposity measures (Table 3; Additional file 14: Figure S5a-d). The genetic risk score based on 29 SNPs showed higher effect estimates per SD increase than our 97 SNPs adult BMI risk score for the childhood adiposity outcomes, especially for preperitoneal fat area (Additional file 8, Table S5). The 97 adult BMI SNPs explained 4.9 % of childhood BMI when added into our model as individual SNPs. When the 97 SNPs were combined into the weighted risk score and added to our model, the risk score explained 1.4 % of childhood BMI (Additional file 15: Table S10).

## Discussion

We observed that a higher overall adult BMI genetic risk score based on 97 SNPs was associated with BMIAP during infancy, and with BMI, total fat mass, android/gynoid fat ratio, and preperitoneal fat area during childhood. A genetic risk score based on SNPs in or close to genes in the membrane proteins pathway was associated with infant PWV, whereas genetic risk scores based on pathways for neuronal developmental processes, hypothalamic processes, cyclicAMP, WNT-signaling, membrane proteins, monogenic obesity/energy homeostasis, glucose homeostasis, cell cycle, and muscle biology were associated with childhood adiposity measures. None of the pathway risk scores were associated with preperitoneal fat area.

### Interpretation of main findings

Previous studies revealed a total of 97 loci related to adult BMI [8]. In a previous study, we reported on the association of a genetic risk score based on 29 adult BMI SNPs known at that time with infant growth and childhood adiposity measures [12]. This risk score was associated with a higher AGEAP and with a higher BMI, total fat mass, android/gynoid fat ratio, and preperitoneal fat area. In the current study, we aimed to identify the effects of updated and more detailed risk scores based on the 97 currently known loci and on subgroups of loci representing specific biological pathways on the same infant growth and childhood adiposity measures. Infant weight growth patterns are known to be strongly associated with BMI in childhood and adulthood, and childhood BMI is associated with obesity and cardiovascular disease in adulthood [1–4, 20, 22, 24]. Thus, it is important to understand the molecular pathways underlying childhood adiposity.

Our results suggest a modest effect of the adult BMI risk score on infant weight growth measures. We observed an association of the overall adult BMI genetic risk score with BMIAP only. In our previous study, based on 29 adult BMI SNPs, the genetic risk score was associated with AGEAP only [12]. A recent study among 9,328 children reported an association of a genetic risk score of 32 adult BMI-associated SNPs, including the 29 included in our previous risk score, with BMIAP, which is in line with our current finding. Additionally, a weak inverse association was found of this risk score with AGEAP [29]. The difference in associations between the previously published 29 SNP adult BMI risk score and our current 97 SNP adult BMI risk score may imply that the increased number of SNPs in the current genetic risk score adds noise to the association of the 97 SNP adult BMI risk score with childhood adiposity outcomes. Also, the analyses were run in a slightly different population, as siblings were excluded for the current study. The added SNPs may be more representative of BMIAP. The childhood BMI genetic risk score was associated with infant PWV and BMIAP, which are both strongly associated with increased risk of overweight in childhood [23]. The overall adult BMI genetic risk score was also associated with all childhood adiposity measures, which is in line with previous studies [12, 29, 30]. Some of these associations are partly explained by infant growth. The WHR risk score was associated with childhood android/gynoid fat ratio only, which is not surprising given the close relation of android/gynoid fat ratio to WHR. Results for the childhood BMI risk score were similar to the associations found with the adult BMI risk score, except that effect estimates were much larger for the child BMI risk score. Larger effect estimates may reflect stronger effects of the childhood-specific SNPs in children. Our results suggest that genetic risk scores based on adult BMI, WHR and childhood BMI influence childhood adiposity outcomes, and also BMI growth patterns from infancy onwards.

The 97 SNPs in our risk score explained 2.7 % of the adult BMI variance in the original paper [8]. In the current study we found that the same SNPs, when added simultaneously to our regression model, account for 4.9 % of childhood BMI suggesting a larger effect of these SNPs in childhood than in adulthood. This may be due to a relative increase in the effects of environmental factors over time. It should be noted that this estimate represents the upper bound of the phenotypic variation accounted for by the 97 SNPs, due to the method of entering all SNPs simultaneously to the model rather than combined into a risk score. When combined into a weighted risk score the 97 SNPs explained only 1.5 % of childhood BMI. We previously reported on a genetic risk score combining only 29 adult BMI SNPs, which explained 2.4 % of the variance in BMI in children of the Generation R Study [12]. Increasing the number of adult SNPs from 29 to 97 thus seemed to add noise to our risk score. It may be that some genetic loci show age-dependent associations with BMI, with different effects in children as compared to adults [31, 32]. Previous work has described an inverse association of the fat mass and obesity related locus (*FTO*) with BMI before the age of 2.5 years, no association between 2.5 and 5 years, and a positive association from around the age of 5 years onwards. The association then strengthens with age, reaching its peak at the age of 20 years and subsequently weakens again [31, 32]. A similar age dependent pattern has been observed for the melanocortin 4 receptor (*MC4R*) locus [32].

Our results showed that during infancy only the membrane proteins pathway affects PWV. This pathway involves membrane proteins that play a role regulating different cell processes involved in e.g., apetite, cholesterol synthesis, and gene expression [8]. Our findings suggest that these processes are also important for weight growth during early life. None of the other pathways were associated with infant growth measures. In line with previous adult studies, a strong role was observed for central nervous system related processes in pathways associated with childhood adiposity measures [8, 33]. Especially the hypothalamic expression and regulation pathway is suggested to be important, which is confirmed in our analyses [8, 31]. Mutations in some of the genes in the monogenic obesity/energy homeostasis related pathways are also suggested to act via central nervous system related processes [33]. The hypothalamic expression and regulation and the monogenic obesity/energy homeostasis pathways were associated with childhood BMI, total fat mass, and android/gynoid fat ratio. The other pathways that were associated with BMI suggest a role for fasting/feeding related processes, glucose homeostasis, signaling, and diabetes related pathways. Our findings suggest a stronger role for the predefined categories during childhood than during infancy, which may be because the childhood measures are more closely related to adult BMI.

Reported total heritability estimates for childhood BMI from twin studies are as high as 80 %. In the current study we found that a risk score based on the known SNPs only explained 1.5 % of the variation in child BMI, emphasizing that a large part of the heritability remains to be discovered [5, 6]. In addition to SNPs, other sources of (epi-) genetic variation, such as copy number variants (CNV) and differences in methylation, may also contribute [34, 35]. A large part of the common CNVs have been efficiently tagged by SNPs in GWA studies [36]. However, associations of rarer CNVs with BMI and obesity showed mixed results [35, 37, 38]. Recently, methylation at specific sites in the DNA has been associated with BMI in adults and children [34, 39, 40]. Additional research in larger study populations is needed to further disentanble the (epi-) genetic background of BMI in children and adults.

### Methodological considerations

The large number of participants and available detailed phenotypes is a major strength of the study. Of all children with genetic data, information on infant growth measures was available for 54 %. Measures of childhood general and abdominal adiposity were available in 72 % of all children. Children without information on infant growth measures had a higher BMI, total fat mass, android/gynoid fat ratio, and preperitoneal fat area (all *p*-values < 0.001) compared with the participants included in our analyses. This may have resulted in an underestimation of the association for the risk scores with infant growth and childhood adiposity measures. Detailed measurements of childhood abdominal adiposity were performed. Both DXA and abdominal ultrasound are considered valid methods for such measurements [24, 26]. Not all SNPs were available in our GWAS dataset. We used a limited number of proxies in very high linkage disequilibrium to complete the sets of SNPs for adult BMI, WHR, and child BMI. No good proxies were available for two WHR SNPs. Given the high number of SNPs available, all risk scores are considered a good representative of the original set of adult SNPs. Although our population is relatively large, we still may have had limited power for these analyses, leaving a possibility of underestimating the number of associated pathways.

## Conclusions

A genetic risk score based on 97 loci associated with adult BMI was associated with PWV during infancy and with general and abdominal fat measurements in childhood. Our results suggest that the genetic background and the pathways involved in adult and childhood adiposity at least partly overlap. Adult BMI related biological pathways involved in neuronal developmental processes, hypothalamic expression and regulation, cyclicAMP, WNT-signaling, membrane proteins, monogenic obesity/energy homeostasis, glucose homeostasis, and cell cycle likely influence adiposity from early life onwards. Further studies are needed to identify more (rare) loci and unravel the underlying mechanisms of childhood adiposity.

## Additional files

Additional file 1: Figure S1. Flow chart of participants. (DOCX 26 kb) Additional file 2: Table S1. List of SNPs included in the main BMI and WHR risk scores. (XLS 23 kb) Additional file 3: Table S2. List of SNPs included per risk score for each biological category. (XLS 34 kb) Additional file 4: Table S3. Matrix of the number of overlapping SNPs per biological category. (XLS 13 kb) Additional file 5: Table S4. Associations of unweighted genetic risk score for BMI with infant growth and childhood adiposity. (DOC 32 kb) Additional file 6: Figure S2. Association of WHR risk score with average peak weight velocity (a), body mass index at adiposity peak (b), and age at adiposity peak (c) (*N* = 2,955). (DOCX 50 kb) Additional file 7: Figure S3. Association of child BMI risk score with average peak weight velocity (a), body mass index at adiposity peak (b), and age at adiposity peak (c) (*N* = 2,955). (DOC 42 kb) Additional file 8: Table S5. Associations of a 29 adult BMI SNPs genetic risk score with infant growth and childhood adiposity. (DOC 31 kb) Additional file 9: Table S6. Phenotypic variance in measures of infant growth explained by genetic risk scores based on adult BMI and WHR SNPs (*N* = 3,975). (DOC 54 kb) Additional file 10: Table S7. Associations of adult BMI genetic risk score with childhood adiposity, additionally adjusted for infant peak weight velocity (*N* = 3,975). (DOC 31 kb) Additional file 11: Table S8. Associations of adult BMI genetic risk score with childhood adiposity, additionally adjusted for BMI at adiposity peak (*N* = 3,975). (DOC 30 kb) Additional file 12: Table S9. Pathway analysis using Ingenuity Pathway Analysis software. (XLS 171 kb) Additional file 13: Figure S4. Association of WHR risk score with average BMI (a), total fat mass (b), android/gynoid fat ratio (c), and preperitoneal fat area (d) (*N* = 3,975). (DOC 47 kb) Additional file 14: Figure S5. Association of child BMI risk score with average BMI (a), total fat mass (b), android/gynoid fat ratio (c), and preperitoneal fat area (d) (*N* = 3,975). (DOC 48 kb) Additional file 15: Table S10. Phenotypic variance in measures of childhood body composition explained by genetic risk scores based on adult BMI and WHR SNPs (*N* = 3,975). (DOC 61 kb)

## Abbreviations

AGEAP: Age at adiposity peak
BMI: Body mass index
BMIAP: Body mass index at adiposity peak
CI: Confidence interval
CNV: Copy number variant
GWAS: Genome-wide association studies
IPA: Ingenuity pathway analysis
PWV: Peak weight velocity
SD: Standard deviation
SDS: Standard deviation scores
SNP: Single nucleotide polymorphism
WHR: Waist-hip ratio
