# Supplementary material for: Associations of genetic risk scores based on adult adiposity pathways with childhood growth and adiposity measures
Source: BMC Genet. 2016 Aug 18;17:120. doi: 10.1186/s12863-016-0425-y (PMC4991119; doi:10.1186/s12863-016-0425-y)
Supplement: Additional file 1: Figure S1. — Flow chart of participants. (DOCX 26 kb) [file 12863_2016_425_MOESM1_ESM.docx]

**Additional file 1: figure S1** Flow chart of participants

Excluded: twin pregnancies
**N= 45**

Data on genetics available
**N= 5,732**

Excluded: no data on at least one outcome measure
**N= 1,536**

Excluded: siblings

**N= 176**

Singleton live births
**N= 5,687**

Data on growth, child anthropometrics and, body composition available

Peak weight velocity Body mass index **N= 2,955 N= 3,975**

BMI at adiposity peak Total fat mass
**N= 2,955 N= 3,798**

Age at adiposity peak Android/gynoid fat ratio **N= 2,955 N= 3,798**

Preperitoneal abdominal fat **N= 3,071**

Data on at least one outcome available
**N= 3,975**
