# Supplementary material for: Associations of genetic risk scores based on adult adiposity pathways with childhood growth and adiposity measures
Source: BMC Genet. 2016 Aug 18;17:120. doi: 10.1186/s12863-016-0425-y (PMC4991119; doi:10.1186/s12863-016-0425-y)
Supplement: Additional file 6: Figure S2. — Association of WHR risk score with average peak weight velocity (a), body mass index at adiposity peak (b), and age at adiposity peak (c) (N = 2,955). (DOCX 50 kb) [file 12863_2016_425_MOESM6_ESM.docx]

**Additional file 6: Figure S2.** Association of WHR risk score with average peak weight velocity (a), body mass index at adiposity peak (b), and age at adiposity peak (c) (N= 2,955)

The x axis represents the categories of the risk score (overall sum of risk alleles, weighted by previous reported effect sizes, rescaled to SDS. The risk score ranged from -4 to 3 SDS and was rounded to the nearest integer for clarity of presentation). The right *y* axis shows the mean SDS and corresponds to the dots. The line represents the regression line of the mean SDS values on the categories of the risk score. The *y* axis on the left corresponds to the histogram representing the number of individuals in each risk-score category. The p-value is based on the continuous risk score, as presented in **Table 2**.
