# Supplementary material for: Associations of genetic risk scores based on adult adiposity pathways with childhood growth and adiposity measures
Source: BMC Genet. 2016 Aug 18;17:120. doi: 10.1186/s12863-016-0425-y (PMC4991119; doi:10.1186/s12863-016-0425-y)
Supplement: Additional file 7: Figure S3. — Association of child BMI risk score with average peak weight velocity (a), body mass index at adiposity peak (b), and age at adiposity peak (c) (N = 2,955). (DOC 42 kb) [file 12863_2016_425_MOESM7_ESM.doc]

**Additional file 7: Figure S3.** Association of child BMI risk score with average peak weight velocity (a), body mass index at adiposity peak (b), and age at adiposity peak (c) (N= 2,955)

The *x* axis represents the categories of the risk score (overall sum of risk alleles, weighted by previous reported effect sizes, rescaled to SDS. The risk score ranged from -4 to 4 SDS and was rounded to the nearest integer for clarity of presentation). The right *y* axis shows mean SDS and corresponds to the dots. The line represents the regression line of the mean SDS values on the categories of the risk score. The *y* axis on the left corresponds to the histogram representing the number of individuals in each risk-score category. The p-value is based on the continuous risk score, as presented in **Table 2**.
