# Supplementary material for: Associations of genetic risk scores based on adult adiposity pathways with childhood growth and adiposity measures
Source: BMC Genet. 2016 Aug 18;17:120. doi: 10.1186/s12863-016-0425-y (PMC4991119; doi:10.1186/s12863-016-0425-y)
Supplement: Additional file 8: Table S5. — Associations of a 29 adult BMI SNPs genetic risk score with infant growth and childhood adiposity. (DOC 31 kb) [file 12863_2016_425_MOESM8_ESM.doc]

**Additional file 8: Table S5. Associations of a 29 adult BMI SNPs genetic risk score with infant growth and childhood adiposity (N= 2,955) ^a,b^**

| **Adult BMI**  **(N=29 SNPs)** | **Peak weight velocity^c^** | **BMI at adiposity peak^c^** | **Age at adiposity peak^c^** | **Body mass index^c,d^** | **Total fat mass^d,e,f^** | **Android/gynoid ratio^d,e,f^** | **Preperitoneal fat area^d,e,f^** |
| --- | --- | --- | --- | --- | --- | --- | --- |
| Beta | 0.011 | 0.031 | 0.032 | 0.149 | 0.121 | 0.109 | 0.070 |
| (95% CI) | (-0.021, 0.042) | (-0.003, 0.065) | (-0.004, 0.069) | (0.121, 0.178) | (0.094, 0.148) | (0.077, 0.140) | (0.037, 0.103) |
| p-value | 0.513 | 0.076 | 0.083 | **1.82*10^-24^** | **6.98*10^-18^** | **2.00*10^-11^** | **2.70*10^-5^** |

^a^Analyses were performed in children with complete data on genetic variants, at least one outcome under study, and covariates. Siblings were excluded for this analysis ^b^Values are linear regression coefficients for models adjusted for sex and the first four genetic principal components and represent the difference in standard deviation scores of the outcome measures for each additional average risk allele in the risk scores. ^d^Values are additionally adjusted for age. ^e^Values are additionally adjusted for height. ^f^Regression coefficients are based on standard deviation scores of ln-transformed outcome measures.
