# Supplementary material for: Associations of genetic risk scores based on adult adiposity pathways with childhood growth and adiposity measures
Source: BMC Genet. 2016 Aug 18;17:120. doi: 10.1186/s12863-016-0425-y (PMC4991119; doi:10.1186/s12863-016-0425-y)
Supplement: Additional file 9: Table S6. — Phenotypic variance in measures of infant growth explained by genetic risk scores based on adult BMI and WHR SNPs (N = 3,975). (DOC 54 kb) [file 12863_2016_425_MOESM9_ESM.doc]

**Additional file 9: Table S6. Phenotypic variance in measures of infant growth explained by genetic risk scores based on adult BMI and WHR SNPs (N= 2,955)**

| **Risk score** | **Peak weight velocity** | **Body mass index at adiposity peak** | **Age at adiposity peak** |  |
| --- | --- | --- | --- | --- |
| **Main risk scores** |  |  |  |  |
| Adult BMI (N=97) | 0.07 | **0.24** | 0.02 |  |
| **Secondary** **risk scores** |  |  |  |  |
| Adult WHR (N=48) | 0.05 | 0.01 | 0.02 |  |
| Child BMI (N=15) | **0.15** | **0.16** | 0.07 |  |
|  |  |  |  |  |
| **Adult BMI pathway genetic risk scores** |  |  |  |  |
| **Neuronal** |  |  |  |  |
| Neuronal Developmental processes (N=29) | 0.122 | 0.219 | 0.034 |  |
| Neurotransmission (N=10) | 0.009 | <0.001 | <0.001 |  |
| Hypothalamic expression and regulatory function (N=13) | <0.001 | 0.007 | 0.054 |  |
| Neuronal Expression (N=12) | 0.118 | 0.010 | 0.066 |  |
| Lipid biosynthesis and metabolism (N=10) | <0.001 | 0.004 | 0.037 |  |
| Bone Development (N=9) | 0.029 | 0.028 | >0.001 |  |
| **Signaling** |  |  |  |  |
| Mitogen activated protein kinase1/Extracellular signal-regulated kinases (N=9) | 0.008 | 0.007 | 0.013 |  |
| JAK (N=2) | 0.005 | 0.002 | 0.003 |  |
| CyclicAMP (N=5) | 0.037 | 0.038 | 0.024 |  |
| WNTSignaling (N=6) | 0.110 | 0.032 | 0.037 |  |
| **GPCR** |  |  |  |  |
| Notch Signaling (N=2) | 0.010 | 0.009 | 0.015 |  |
| Mitochondrial (N=8) | 0.009 | 0.001 | 0.132 |  |
| Retinoic Acid Receptors (N=6) | 0.036 | 0.065 | 0.034 |  |
| Endocytosis/Exocytosis (N=14) | 0.002 | 0.002 | 0.005 |  |
| Eye-related (N=5) | 0.010 | 0.010 | 0.082 |  |
| Tumorigenesis (N=11) | 0.030 | 0.030 | >0.001 |  |
| Apoptosis (N=13) | 0.077 | 0.034 | 0.104 |  |
| Membrane Proteins (N=12) | **0.330** | 0.243 | 0.078 |  |
| Hormone metabolism/regulation (N=4) | 0.009 | 0.008 | 0.009 |  |
| Purine/Pyrimidine cycle (N=4) | 0.009 | 0.159 | 0.060 |  |
| Monogenic Obesity and/or Energy Homeostasis (N=9) | 0.018 | 0.021 | 0.063 |  |
| Immune System (N=15) | 0.210 | 0.250 | 0.001 |  |
| Limb Development (N=3) | 0.032 | 0.219 | >0.001 |  |
| Ubiquitin pathways (N=6) | 0.004 | 0.005 | 0.063 |  |
| Glucose homeostasis and/or diabetes (N=11) | 0.052 | 0.046 | 0.067 |  |
| Cell cycle (N=23) | 0.007 | 0.012 | >0.001 |  |
| **DNARepair** |  |  |  |  |
| Nuclear trafficking (N=4) | 0.022 | 0.053 | 0.095 |  |
| Muscle Biology (N=6) | 0.013 | >0.001 | 0.019 |  |

Bold values represent explained variances for significant associations of the risk score with the outcome.
