# Supplementary material for: Associations of genetic risk scores based on adult adiposity pathways with childhood growth and adiposity measures
Source: BMC Genet. 2016 Aug 18;17:120. doi: 10.1186/s12863-016-0425-y (PMC4991119; doi:10.1186/s12863-016-0425-y)
Supplement: Additional file 11: Table S8. — Associations of adult BMI genetic risk score with childhood adiposity, additionally adjusted for BMI at adiposity peak (N = 3,975). (DOC 30 kb) [file 12863_2016_425_MOESM11_ESM.doc]

**Additional file 11: Table S8. Associations of adult BMI genetic risk score with childhood adiposity, additionally adjusted for BMI at adiposity peak (N= 3,975)^a, b^**

| **Risk score** | **Body mass index^c,d^** |  | **Total fat mass^d,e,f^** |  | **Android/gynoid ratio^d,e,f^** |  | **Preperitoneal fat area^d,e,f^** |  |
| --- | --- | --- | --- | --- | --- | --- | --- | --- |
| **(number of SNPs in risk score)** | **Beta (CI 95%)** | **P-value** | **Beta (CI 95%)** | **P-value** | **Beta (CI 95%)** | **P-value** | **Beta (CI 95%)** | **P-value** |
| Adult BMI (N=97) | 0.095 (0.066; 0.123) | **4.76*10^-11^** | 0.080 (0.050; 0.109) | **1.78*10^-7^** | 0.075 (0.040; 0.109) | **3.00*10^-5^** | 0.015 (-0.022; 0.051) | 0.430 |
| Child BMI (N=15) | 0.070 (0.042; 0.098) | **9.33*10^-7^** | 0.061 (0.031; 0.090) | **7.00*10^-5^** | 0.076 (0.041; 0.111) | **1.90*10^-5^** | 0.033 (-0.003; 0.069) | 0.073 |

^a^Analyses were performed in children with complete data on genetic variants, at least one outcome under study, and covariates ^b^Values are linear regression coefficients for models adjusted for sex, BMI at adiposity peak, and the first four genetic principal components and represent the difference in standard deviation scores of the outcome measures for each additional average risk allele in the risk scores. ^d^Values are additionally adjusted for age. ^e^Values are additionally adjusted for height. ^f^Regression coefficients are based on standard deviation scores of ln-transformed outcome measures.
