# Supplementary material for: Associations of genetic risk scores based on adult adiposity pathways with childhood growth and adiposity measures
Source: BMC Genet. 2016 Aug 18;17:120. doi: 10.1186/s12863-016-0425-y (PMC4991119; doi:10.1186/s12863-016-0425-y)
Supplement: Additional file 14: Figure S5. — Association of child BMI risk score with average BMI (a), total fat mass (b), android/gynoid fat ratio (c), and preperitoneal fat area (d) (N = 3,975). (DOC 48 kb) [file 12863_2016_425_MOESM14_ESM.doc]

**Additional file 14: Figure S5.** Association of child BMI risk score with average BMI (a), total fat mass (b), android/gynoid fat ratio (c), and preperitoneal fat area (d) (N= 3,975)

The *x* axis represents the categories of the risk score (overall sum of risk alleles, weighted by previous reported effect sizes, rescaled to SD-scores. The risk score ranged from -4 to 4 SDS and was rounded to the nearest integer for clarity of presentation). The right *y* axis shows the mean SDS and corresponds to the dots and a line representing the regression line of the mean SDS values for each category of the risk score. The *y* axis on the left corresponds to the histogram representing the number of individuals in each risk-score category. The p-value is based on the continuous risk score, as presented in **Table 3**.
