# Supplementary material for: Associations of genetic risk scores based on adult adiposity pathways with childhood growth and adiposity measures
Source: BMC Genet. 2016 Aug 18;17:120. doi: 10.1186/s12863-016-0425-y (PMC4991119; doi:10.1186/s12863-016-0425-y)
Supplement: Additional file 15: Table S10. — Phenotypic variance in measures of childhood body composition explained by genetic risk scores based on adult BMI and WHR SNPs (N = 3,975). (DOC 61 kb) [file 12863_2016_425_MOESM15_ESM.doc]

**Additional file 15: Table S10.** Phenotypic variance in measures of childhood body composition explained by genetic risk scores based on adult BMI and WHR SNPs (N= 3,975).

| **Risk score** | **Body mass index** | **Total fat mass** | **Android/gynoid ratio** | **Preperitoneal fat area** |
| --- | --- | --- | --- | --- |
| **Main risk scores** |  |  |  |  |
| Adult BMI (N=97) | **1.43** | **0.81** | **0.57** | **0.11** |
| **Secondary** **risk scores** |  |  |  |  |
| Adult WHR (N=48) | 0.02 | 0.01 | **0.50** | 0.08 |
| Child BMI (N=15) | **0.95** | **0.52** | **0.64** | **0.14** |
| **Risk scores based on 97 adult BMI** |  |  |  |  |
| **Neuronal** |  |  |  |  |
| Neuronal Developmental processes (N=29) | **0.328** | 0.087 | 0.119 | 0.005 |
| Neurotransmission (N=10) | 0.019 | 0.001 | 0.001 | 0.007 |
| Hypothalamic expression and regulatory function (N=13) | **1.136** | **0.767** | **0.626** | 0.159 |
| Neuronal Expression (N=12) | 0.033 | 0.036 | 0.123 | 0.008 |
| Lipid biosynthesis and metabolism (N=10) | 0.061 | 0.017 | 0.025 | <0.001 |
| Bone Development (N=9) | 0.035 | 0.004 | 0.023 | 0.002 |
| **Signaling** |  |  |  |  |
| Mitogen activated protein kinase1/Extracellular signal-regulated kinases (N=9) | 0.135 | 0.132 | 0.052 | 0.020 |
| JAK (N=2) | 0.125 | 0.039 | 0.014 | 0.005 |
| CyclicAMP (N=5) | 0.229 | **0.247** | 0.138 | 0.061 |
| WNTSignaling (N=6) | **0.387** | 0.083 | 0.145 | 0.101 |
| **GPCR** |  |  |  |  |
| Notch Signaling (N=2) | 0.086 | 0.074 | 0.079 | 0.079 |
| Mitochondrial (N=8) | **0.165** | 0.144 | <0.001 | 0.001 |
| Retinoic Acid Receptors (N=6) | 0.232 | 0.136 | 0.025 | 0.029 |
| Endocytosis/Exocytosis (N=14) | 0.017 | 0.001 | 0.045 | 0.039 |
| Eye-related (N=5) | 0.017 | 0.022 | 0.014 | 0.001 |
| Tumorigenesis (N=11) | 0.180 | 0.037 | 0.025 | 0.016 |
| Apoptosis (N=13) | 0.072 | 0.038 | 0.006 | 0.124 |
| Membrane Proteins (N=12) | **0.640** | **0.187** | **0.346** | 0.012 |
| Hormone metabolism/regulation (N=4) | 0.047 | 0.175 | 0.066 | 0.002 |
| Purine/Pyrimidine cycle (N=4) | 0.019 | 0.002 | 0.029 | 0.005 |
| Monogenic Obesity and/or Energy Homeostasis (N=9) | **0.610** | **0.442** | **0.410** | 0.084 |
| Immune System (N=15) | 0.235 | 0.133 | 0.042 | 0.006 |
| Limb Development (N=3) | 0.142 | 0.044 | 0.079 | 0.002 |
| Ubiquitin pathways (N=6) | 0.006 | 0.004 | 0.012 | 0.022 |
| Glucose homeostasis and/or diabetes (N=11) | **0.282** | 0.052 | 0.173 | 0.008 |
| Cell cycle (N=23) | 0.226 | **0.210** | 0.056 | 0.004 |
| **DNARepair** |  |  |  |  |
| Nuclear trafficking (N=4) | 0.003 | 0.008 | 0.002 | 0.007 |
| Muscle Biology (N=6) | **0.262** | 0.081 | 0.058 | 0.046 |

Bold values represent explained variances for significant associations of the risk score with the outcome.
